# Complete Chloroplast Genome of *Sedum sarmentosum* and Chloroplast Genome Evolution in Saxifragales

**DOI:** 10.1371/journal.pone.0077965

**Published:** 2013-10-18

**Authors:** Wenpan Dong, Chao Xu, Tao Cheng, Shiliang Zhou

**Affiliations:** 1 State Key Laboratory of Systematic and Evolutionary Botany, Institute of Botany, Chinese Academy of Sciences, Beijing, China; 2 Graduate University of Chinese Academy of Sciences, Beijing, China; University of Lausanne, Switzerland

## Abstract

Comparative chloroplast genome analyses are mostly carried out at lower taxonomic levels, such as the family and genus levels. At higher taxonomic levels, chloroplast genomes are generally used to reconstruct phylogenies. However, little attention has been paid to chloroplast genome evolution within orders. Here, we present the chloroplast genome of *Sedum sarmentosum* and take advantage of several available (or elucidated) chloroplast genomes to examine the evolution of chloroplast genomes in Saxifragales. The chloroplast genome of *S. sarmentosum* is 150,448 bp long and includes 82,212 bp of a large single-copy (LSC) region, 16.670 bp of a small single-copy (SSC) region, and a pair of 25,783 bp sequences of inverted repeats (IRs).The genome contains 131 unique genes, 18 of which are duplicated within the IRs. Based on a comparative analysis of chloroplast genomes from four representative Saxifragales families, we observed two gene losses and two pseudogenes in *Paeonia obovata*, and the loss of an intron was detected in the *rps16* gene of *Penthorum chinense*. Comparisons among the 72 common protein-coding genes confirmed that the chloroplast genomes of *S. sarmentosum* and *Paeonia obovata* exhibit accelerated sequence evolution. Furthermore, a strong correlation was observed between the rates of genome evolution and genome size. The detected genome size variations are predominantly caused by the length of intergenic spacers, rather than losses of genes and introns, gene pseudogenization or IR expansion or contraction. The genome sizes of these species are negatively correlated with nucleotide substitution rates. Species with shorter duration of the life cycle tend to exhibit shorter chloroplast genomes than those with longer life cycles.

## Introduction

Chloroplasts are one of the main distinctive characteristics of plant cells. The major function of chloroplasts is to perform photosynthesis [1]. Typically, the size of chloroplast genomes in higher plants ranges from 120 to 160 kb, and a pair of inverted repeats (IRs) divides the genome into a large single copy (LSC) region and a small single copy (SSC) region. Most chloroplast genomes contain 110–130 distinct genes; the majority of these genes (approximately 79) encode proteins, which are mostly involved in photosynthesis, while the remainder of the genes encode transfer RNAs (approximately 30) or ribosomal RNAs (4) [2]. 

Since the first chloroplast genome from tobacco (*Nicotiana tabacum*) was published [3], more than 200 complete chloroplast genomes from protists, thallophytic, bryophytic, and vascular plants have been made available in GenBank. Although the chloroplast genomes of vascular plants are highly conserved in their basic structures, comparative genomic studies have revealed occasional structural changes, such as inversions, gene or intron losses, and rearrangements among plant lineages. The most notable examples of gene loss were found in the parasitic plants *Cuscuta* [4,5], *Epifagus* [6], and *Rhizanthella* [7], which have lost some or all of their photosynthetic ability. Loss of chloroplast genes is rare in photosynthetic species but can occur if a gene has been transferred to the nuclear genome or functionally replaced by a nuclear gene [8]. For instance, the *rpl22* gene of Fagaceae and Passifloraceae [9], the *infA* gene of Brassicaceae [10], and the *rpl32* gene of *Populus* [11] have transferred to the nuclear genome. Only 18 genes found in angiosperm chloroplast genomes contain introns, and most of them are quite conserved. However, the introns of the *rpoC1*, *rpl2*, and *atpF* genes have been independently lost from the chloroplast genomes of some angiosperm lineages [10,12-15]. Extensions or contractions of IR regions that cause variations in genome size, together with gene losses and nucleotide insertions/deletions (indels), are frequently observed within intergenic spacers [16].

**Figure 1:**
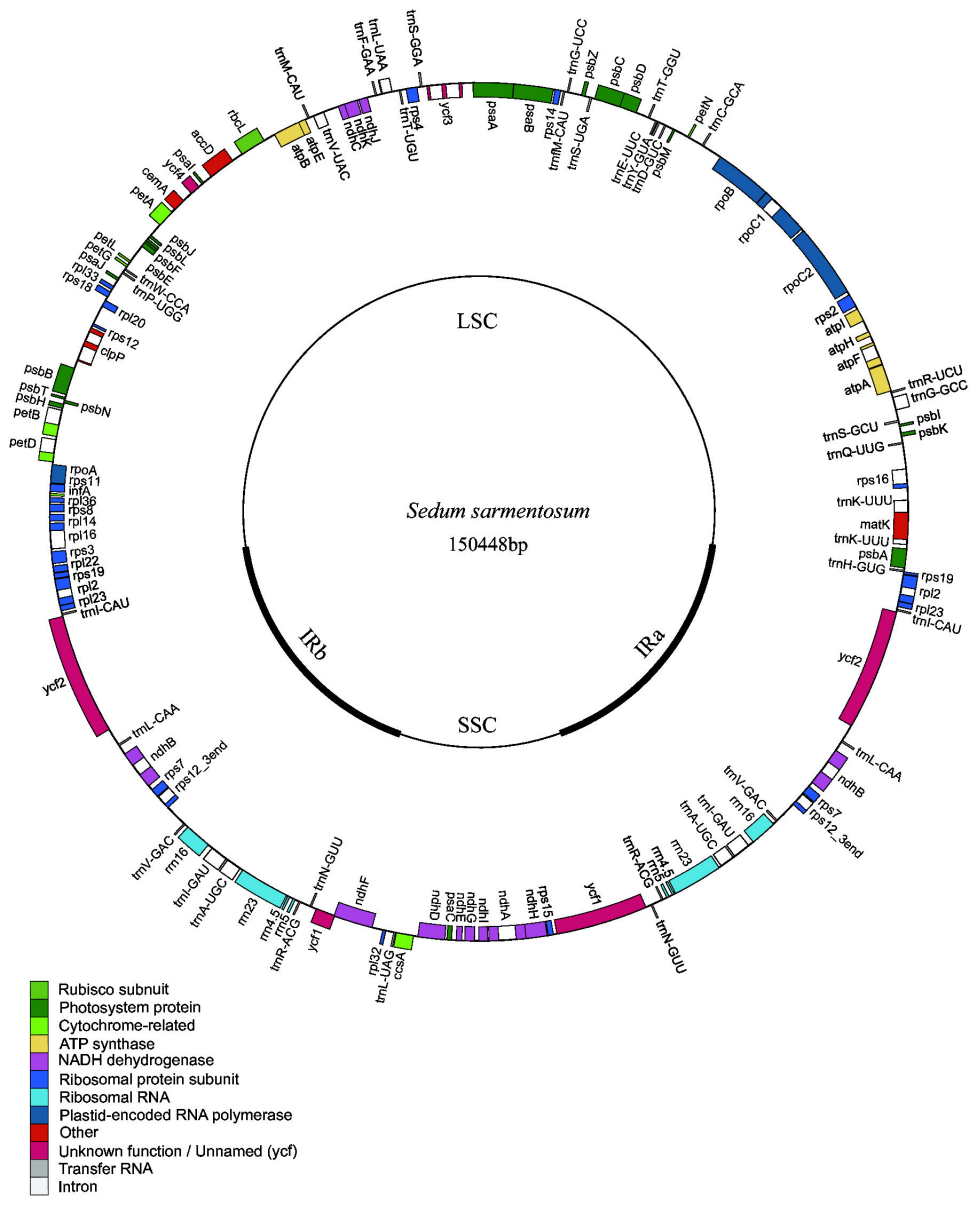
Chloroplast genome map of *Sedum sarmentosum*. The genes inside and outside of the circle are transcribed in the clockwise and counterclockwise directions, respectively. Genes belonging to different functional groups are shown in different colors. The thick lines indicate the extent of the inverted repeats (IRa and IRb) that separate the genomes into small single-copy (SSC) and large single-copy (LSC) regions.

The nucleotide substitution rate of chloroplast genes is lower than that of nuclear genes but higher than that of mitochondrial genes [17]. “The overall relative rate of synonymous substitutions of mitochondrial, chloroplast, and nuclear genes in all seed plants is 1:3:10” [18]. However, the rate of chloroplast genome evolution appears to be taxon and gene dependent. For example, the substitution rates observed in the chloroplast genomes of gnetophytes are significantly higher than in other gymnosperms [19,20]; the Poaceae have experienced accelerated chloroplast genome rearrangements and nucleotide substitutions compared to other monocots [14]; and the genes encoding ribosomal proteins, RNA polymerase, and ATPase in Geraniaceae undergo nucleotide substitutions more rapidly than photosynthetic genes [21]. 

Considering its small size, simple structure and conserved gene content, the chloroplast genome has become an ideal model for evolutionary and comparative genomic researches. Comparative studies of chloroplast genomes have mostly been focused on a target species such as *Panicum virgatum* [22]; genera such as *Oenothera* [23,24]; or families such as Solanaceae [25,26], Poaceae [27,28], Pinaceae [29,30], and Asteraceae [31]. At higher taxonomic levels, information on chloroplast genomes is useful not only for phylogenetic studies [10,32,33] but also for understanding the genome evolution underlying gene and intron losses, genome size variations, and nucleotide substitutions. For this purpose, Saxifragales is an ideal group, in which four completely sequenced and one nearly completely sequenced chloroplast genomes are available, representing five major lineages in the order, i.e., the woody lineage, Haloragaceae + Penthoraceae, Crassulaceae, the Saxifragaceae alliance and the fence-riding Paeoniaceae [34].

As defined in the APG III system (2009), Saxifragales includes 15 families and is divided into six major lineages [35]. The Saxifragales are morphologically diverse, including herbs, shrubs, and large trees [34]. Saxifragales represents one of the early diversified orders of rosids. It was estimated that the order has diverged from eurosids for 89.1−97.6 million years and all major lineages had diverged one another in Cretaceous [36]. It would be interesting to know what has happened to their chloroplast genomes after such a long time of evolution. In Saxifragales the complete chloroplast genome of *Liquidambar formosana* (Hamamelidaceae) and the protein-coding genes of *Heuchera sanguinea* (Saxifragaceae) have been reported [32], and the genomes of *Paeonia obovata* (Paeoniaceae) and *Penthorum chinense* (Penthoraceae) have also been determined (They will be published soon in another paper). If a chloroplast genome of Crassulaceae is available, we will be able to probe into the chloroplast genome evolution in the order. Thus, we chose to determine the chloroplast genome of *Sedum sarmentosum* (Crassulaceae) which is a frequently observed herbal ornamental plant with some medicinal values. 

Here, we first report the complete sequence of the chloroplast genome of *S. sarmentosum*. Then, we present the results of comparative analyses of four representative chloroplast genomes of Saxifragales species, using the genome of the closely related but basal species *Vitis vinifera* as a reference. Special emphases were placed on changes in genome structure, the variations of nucleotide substitution rates and genome sizes, and the associations between them.

## Materials and Methods

### DNA extraction and sequencing

Genomic DNA was extracted from fresh young leaves of an *S. sarmentosum* plant found in the Beijing Botanical Garden (Institute of Botany, Chinese Academy of Sciences, Beijing, China) using the mCTAB method [37]. The genome was sequenced following Dong et al. [35]. Fifty-five specific primers (Table S1) were used to bridge gaps in the chloroplast genome.

### Chloroplast genome annotation

Genome annotation was accomplished using the Dual Organellar Genome Annotator (DOGMA) [38] to annotate the genes encoding proteins, transfer RNAs (tRNAs), and ribosomal RNAs (rRNAs). All of the identified tRNA genes were further verified using the corresponding structures predicted by tRNAscan-SE 1.21 [39].

### Comparative chloroplast genomic analysis

The mVISTA program was employed in Shuffle-LAGAN mode [40] to compare the complete chloroplast genomes of *S. sarmentosum* and three other species (*Liquidambar formosana*, *Penthorum chinense*, and *Paeonia obovata*). The chloroplast genome of *Vitis vinifera* was used as a reference. To assess the variability of the coding regions of the four chloroplast genomes together with that of *Heuchera sanguinea* (Saxifragaceae) [32], the nucleotide sequences of all protein-coding genes were aligned to those of the reference genome using ClustalX [41] and adjusted manually using Se-Al 2.0 [42]. 

### Estimation of substitution rates

The relative rates of sequence divergence in the five Saxifragales species and the reference were analyzed using the PAML v4.4 package [43]. The program yn00 was employed to estimate dN, dS, and dN/dS under the F3x4 substitution matrix using the Nei–Gojobori method. Genes with the same functions were grouped following previous studies [44-46]. Analyses were carried out on (1) concatenated common protein-coding genes, except for lost genes or pseudogenes from any species; 2) datasets corresponding to the same functions, i.e., for *atp*, *pet*, *ndh*, *psa*, *psb*, *rpl*, *rpo*, and *rps*; and 3) datasets corresponding to singular genes, i.e., for *cemA*, *matK*, *ccsA*, *clpP*, *rbcL*, and *ycf1*. Tajima’s relative rate test implemented in MEGA 5 was used to compare evolutionary rates among the lineages [47,48]. Kruskal-Wallis and Spearman’s rank correlation tests were conducted using the R software package (http://www.r-project.org) 

**Figure 2:**
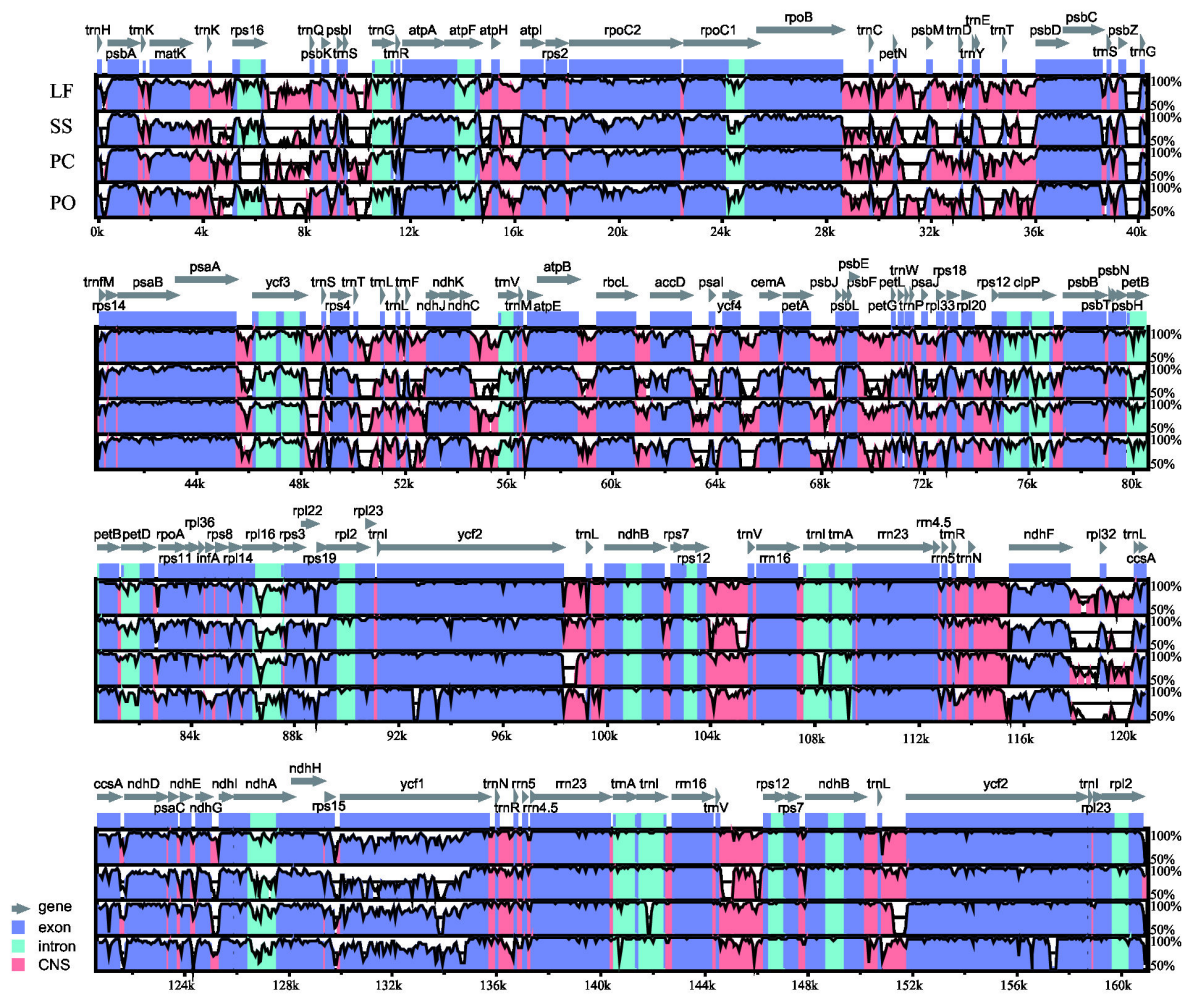
Identity plot comparing the chloroplast genomes of four Saxifragales species using *Vitis vinifera* as a reference sequence. The vertical scale indicates the percentage of identity, ranging from 50% to 100%. The horizontal axis indicates the coordinates within the chloroplast genome. Genome regions are color coded as protein-coding, rRNA, tRNA, intron, and conserved non-coding sequences (CNS). Abbreviations - LF: *Liquidambar formosana*; SS: *Sedum sarmentosum*; PC: *Penthorum chinense*; and PO: *Paeonia obovata*.

## Results and Discussion

### Genome content and organization in *Sedum sarmentosum*

The complete chloroplast genome of *S. sarmentosum* (JX427551) is 150,448 bp in size and exhibits a typical circular structure including a pair of IRs (25,783 bp each) that separate the genome into two single-copy regions (LSC 82,212 bp; SSC 16,670 bp; Figure 1). Coding regions (91,260 bp), including protein-coding genes (79,413 bp), tRNA genes (2,801 bp), and rRNA genes (9,046 bp), account for 60.66% of the genome, while noncoding regions (59,188 bp), including introns (17,750 bp) and intergenic spacers (41,438 bp), account for the remaining 39.34% of the genome. The overall A+T content of the whole genome is 62.24% (Table 1).

**Figure 3:**
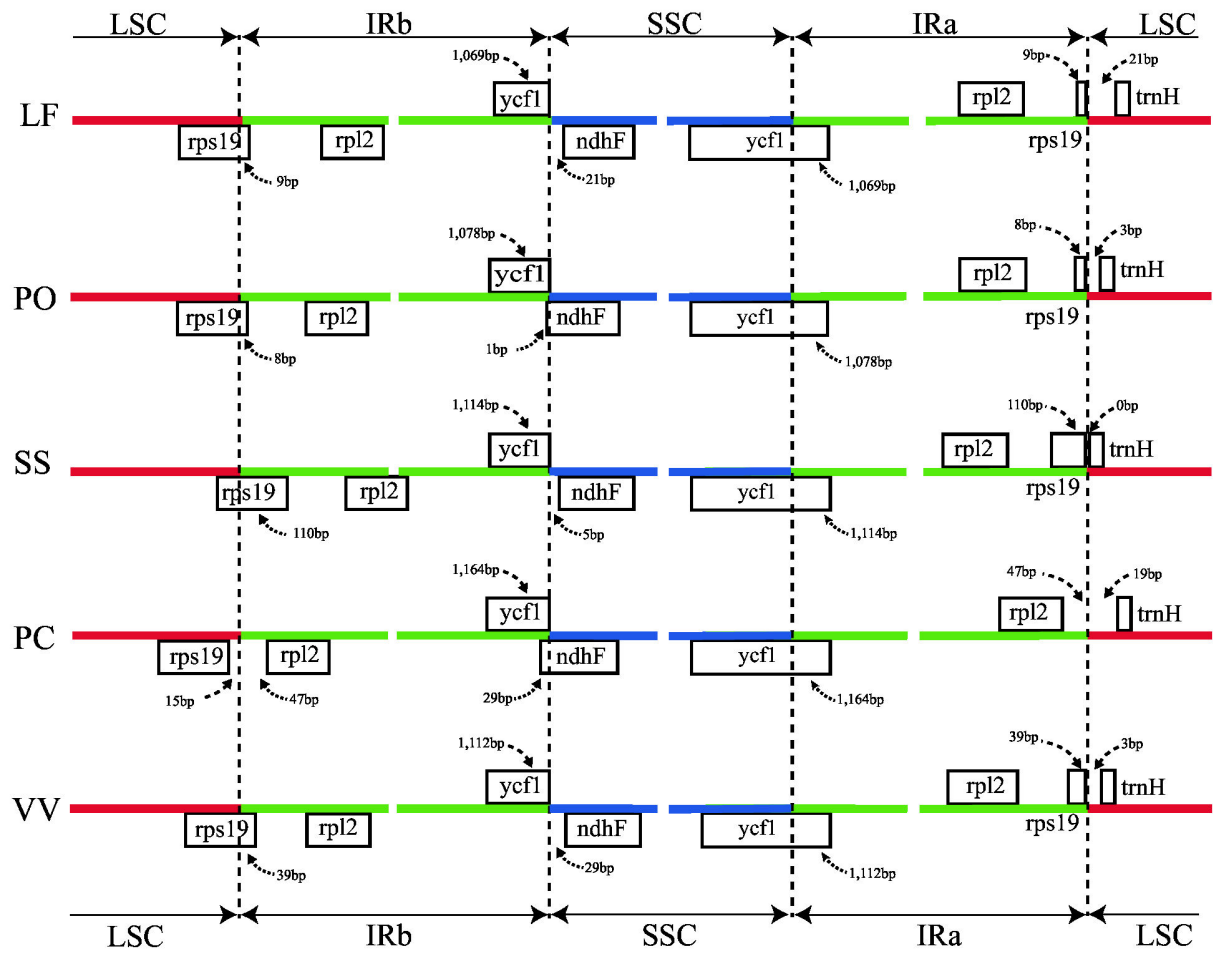
Comparison of junction positions between single copy and IR regions among four Saxifragales genomes and *Vitis vinifera*. Abbreviations - LF: *Liquidambar formosana*; PO: *Paeonia obovata*; SS: *Sedum sarmentosum*; PC: *Penthorum chinense*; and VV: *Vitis vinifera*.

**Table 1:** Characteristics of four Saxifragales and an outgroup (*Vitis vinifera*) chloroplast genomes.

Features	*Sedum sarmentosum*	*Paeonia obovata*	*Penthorum chinense*	*Liquidambar formosana*	*Vitis vinifera*
Genome size	150448	152736	156686	160410	160928
Length of LSC	82212	84399	86735	88945	89147
Length of SSC	16670	17031	20399	18917	19065
Length of IR	25783	25653	25776	26274	26358
Coding size	91260	89941	91756	91840	90878
Intron size^1^	17750	17461	16380	17216	17977
Spacer size	41438	45334	48550	51354	52073
AT content (%)	62.24%	61.57%	62.73%	62.05%	62.20%
Total number of genes	131	127	131	131	131
Protein-coding genes	79	75	79	79	79
Duplicated genes	18	18	18	18	18
tRNA genes	30	30	30	30	30
rRNA genes	4	4	4	4	4
Genes with introns	18	18	17	18	18
Pseudogenes	1	3	1	1	1

^1^ The *trnK* intron is not considered.

There are a total of 113 genes in the genome, including 79 protein-coding genes, 30 tRNA genes, and 4 ribosomal RNA genes (Figure 1 and Table S2). Eighteen genes contain introns (one class I intron, *trnL* ^*UUA*^, and 17 class II introns), and three of these genes, *clpP*, *rps12*, and *ycf3*, exhibit two introns. The 5′-end exon of the *rps12* gene is located in the LSC region, and the intron and 3′-end exon of the gene are situated in the IR region. 

**Table 2:** Substitution rates of 72 protein-coding genes in five Saxifragales chloroplast genomes.

Taxa	Nonsynonymous (dN)	Synonymous (dS)	dN/dS
*Liquidambar formosana*	0.0269±0.0007	0.1234±0.0032	0.2178
*Heuchera sanguinea*	0.0299±0.0008	0.1480±0.0035	0.2019
*Penthorum chinense*	0.0314±0.0008	0.1590±0.0037	0.1975
*Paeonia obovata*	0.0422±0.0009	0.2358±0.0048	0.1788
*Sedum sarmentosum*	0.0609±0.0011	0.2931±0.0056	0.2077

*Vitis vinifera* was used as an outgroup. Data are presented as the means ± standard error.

### Genome organization of Saxifragales

The organization of the chloroplast genome is rather conserved within Saxifragales (Figure 2). Neither translocations nor inversions were detected in the four Saxifragales genomes. Similar to other angiosperms, the IR region is more conserved in these species than the LSC and SSC regions. Differences were observed in terms of genome size, gene losses, intron losses, the pseudogenization of protein-coding genes, and IR expansion and contraction.

**Figure 4:**
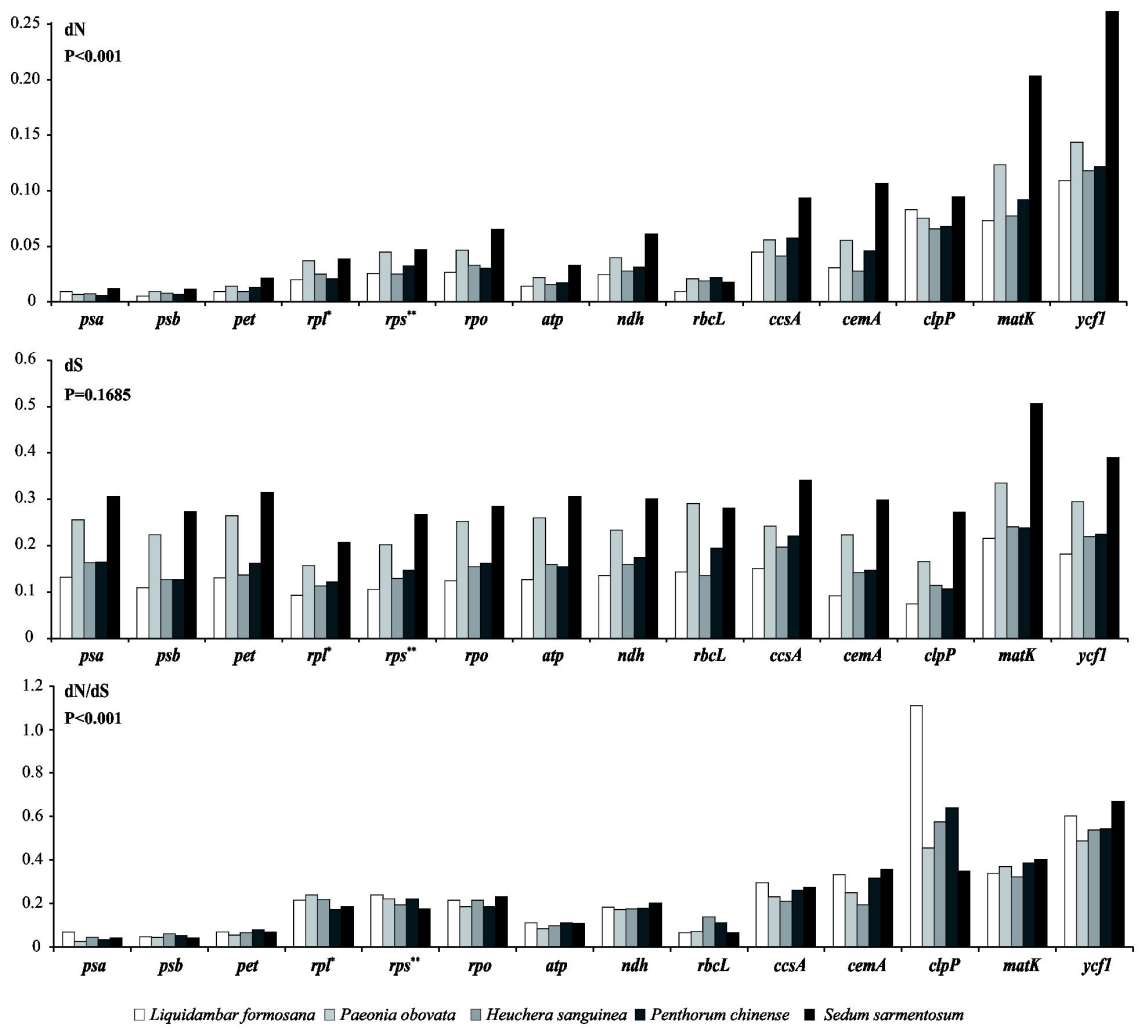
Nonsynonymous substitution (dN), synonymous substitution (dS), and dN/dS values for individual Saxifragales genes and groups of genes. *Without the *rpl22* and *rpl32* genes; **without the *rps18* gene.

#### Genome size

In terms of the chloroplast genome size observed among the examined Saxifragales species, *S.* sarmentosum exhibits the smallest genome. The genome of *L. formosana* (160,410 bp) is approximately 10 kbp larger than that of *S. sarmentosum*, 6.2 kbp larger than that of *Penthorum chinense*, and 2.7 kbp larger than that of *Paeonia obovata*, though it is 0.5 kbp smaller than that of *V. vinifera*, an out-group species. The detected sequence length variation is mainly attributable to the difference in the length of the noncoding region (Table 1). The *S. sarmentosum* genome contains the smallest noncoding region among the four analyzed chloroplast genomes. 

#### Gene loss

Two genes, *infA* and *rpl32*, have been lost from the chloroplast genome of *Paeonia obovata*. The only case of gene loss observed in the sampled Saxifragales species was for *infA* which functions as a translation initiation factor that assists in the assembly of the translation initiation complex [49]. Loss of *infA* appears to have independently occurred multiple times during the evolution of land plants, and this gene is also possibly transferred to the nucleus [50]. Therefore, its loss in *Paeonia obovata* does not represent a unique phenomenon. The *rpl32* gene is one of the nine genes encoding the large ribosomal protein subunit, and loss of this gene would cause functional problems. In *Populus*, the *rpl32* gene has been transferred to the nucleus [11]. In *Paeonia obovata*, whether the *rpl32* gene has been transferred to the nucleus remains to be investigated. 

#### Intron loss

The *rps16* gene in the chloroplast genome of *Penthorum chinense* has lost its only intron, which is a phenomenon that is also observed in *Trachelium* [51] and *Pelargonium* [52]. However, the genome of *Penthorum chinense* is conventional, whereas those of *Trachelium* and *Pelargonium* have been extensively restructured. The intron loss observed for *rps16* is therefore unusual in normal angiospermous chloroplast genomes.

**Figure 5:**
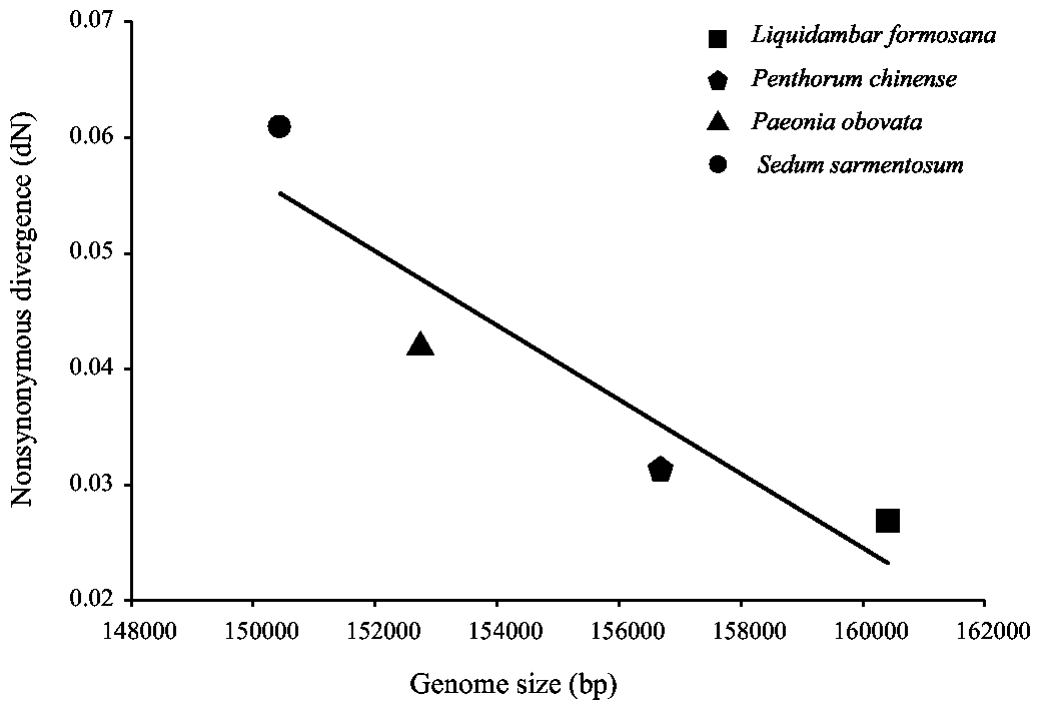
Negative correlation between nonsynonymous substitution (dN) values and chloroplast genome size. The nonsynonymous substitution values are based on an analysis of all protein-coding genes, except for *rpl32*, *infA*, *rpl22*, and *rps18*.

The *rpl2* intron loss has been reported in Saxifragaceae genera, including *Saxifraga* and *Heuchera* [13]. This phenomenon was confirmed in *Heuchera sanguinea* (GenBank accession number: GQ998409; HQ664603) but rejected in *Heuchera micrantha* (GenBank accession number: EF207446) and *Saxifraga stolonifera* (GenBank accession number: EF207457) based on a re-examination of the sequences. The *rpl2* intron is present in all of the Saxifragales genomes examined in this study, suggesting that intron loss in the *rpl2* gene is occasional.

#### Gene pseudogenization

The *rpl22* and *rps18* are pseudogenes only in Paeoniaceae, but *ycf15* has pseudogenized in all families of Saxifragales. The *rpl22* sequence of *Paeonia obovata* contains an insertion of a C residue at position 236, which causes a reading frame shift and internal stop codons (Figure S1). The *rps18* gene of *Paeonia obovata* exhibits a mutation at the 58th codon and a deletion of a single nucleotide at the 86th codon (Figure S2). The *rps18* gene encodes a small ribosomal protein and has only been found lost or pseudogenized in the chloroplast genomes of some parasitic plants [49]. 

The *ycf15* gene, which displays a small open reading frame (ORF), is located immediately downstream of the *ycf2* gene. The *ycf15* gene of tobacco is potentially functional [3], but the validity of *ycf15* as a protein-coding gene is questionable [53-55]. Expression studies in spinach have suggested that *ycf15* may be transcribed, but not spliced [56]. The *ycf15* is certainly a pseudogene in Saxifragales. There is a deletion of ~400 bp in *Penthorum chinense* (Figure S3) and an inversion of 15 bp in *S. sarmentosum*. Although the extra sequences (compared to *Nicotiana*) found in *S. sarmentosum* and the other three species were ignored, the *ycf15* genes of these species exhibit premature stop codons. Because small inversions and microstructural changes mostly occur in introns and intergenic spacers [57], the extra sequence of *ycf15* is more appropriately considered an intron of a pseudogene. 

#### IR expansion and contraction

The expansion and contraction of the border regions between the two IR regions and the single-copy regions have contributed to genome size variations among plant lineages [58]. Therefore, we compared the exact IR border positions and their adjacent genes among the four Saxifragales chloroplast genomes (Figure 3). The portion of *ycf1* located in the IR region varies from 1069 bp to 1164 bp. The *ndhF* gene shares some nucleotides with the *ycf1* pseudogene (1 bp in *Paeonia obovata* and 29 bp in *Penthorum chinense*) or is separated from *ycf1* by spacers (5 bp in *S. sarmentosum*, 26 bp in *L. formosana* and 29 bp in *V. vinifera*). 

The IRa/LSC border is generally located upstream of the *trnH* ^*GUG*^ gene. The *trnH* ^*GUG*^ gene is separated from the *rps19* pseudogene or the *rpl2* gene by a spacer except in *S. sarmentosum* which does not contain a spacer (Figure 3). However, in *Penthorum chinense*, the *rps19* gene does not extend into the IR region, and thus, the *rps19* pseudogene is not observed. Although there are expansions or contractions of IR regions observed among the sampled representatives of Saxifragales, they contribute little to the overall size differences in the chloroplast genomes.

### Genome evolution

Nonsynonymous (dN) and synonymous (dS) substitutions and their ratio (ω=dN/dS) are indicators of the rates of evolution and natural selection [59]. Relative to the reference species *V. vinifera*, these parameters (Table 2) were compared among the protein-coding chloroplast genes of the five representative species of Saxifragales to investigate genome evolution. The dN and dS of *S. sarmentosum* are 2.27 fold and 2.3 fold larger than those of *L. formosana*, respectively, whereas the values of *Paeonia obovata* are accordingly 1.56 fold and 1.88 fold higher than those of *L. formosana*. The ω values of these Saxifragales species are significantly smaller than 1, suggesting the existence of purifying selection on the chloroplast protein-coding genes of Saxifragales species. Both the dN and dS values also consistently indicated that *Paeonia obovata* and *S. sarmentosum* have evolved significantly more rapidly than the other three species (χ^2^-test, all P <0.001). 

#### Association of dN with gene functions

Variations in evolutionary rates can be related to genome structure and the function of genes [10,44]. In Saxifragales species, the observed genome structures are quite similar, without any remarkable restructuring being detected. In comparison with the out-group *V. vinifera*, the dS shows similar values (Kruskal–Wallis tests, P = 0.1685), whereas the dN values differ significantly (P<0.001) among gene groups sorted according to gene functions (Figure 4). The *psa*, *psb*, and *pet* genes exhibit the lowest dN values, while the *ycf1* gene presents the highest dN values. The *matK*, *clpP*, *ccsA*, and *cemA* genes have evolved more rapidly than the other gene groups in Saxifragales. Apart from individual genes, *rpo* exhorted the highest mean dN value, followed by *ndh*, *rps*, and *rpl*. Moreover, a strong positive correlation (Spearman’s rank correlation, r_S_ = 0.94, P < 0.001) was observed between dN and dS among genes of ribosomal gene groups (*rpl*, *rpo* and *rps*), but no significant correlation was found between these two values for any other gene groups. 

#### Accelerated evolution of the *S. sarmentosum* and *Paeonia obovata* genomes

Tajima’s relative rate tests of dN strongly (P < 0.001) suggest an acceleration of nucleotide substitution rates in the genomes of *S. sarmentosum* and *Paeonia obovata*. These two genomes are similar to others in terms of their genome structure and number of genes and appear to be under the same purifying selection, but they differ from other species in terms of their genome size, life histories and the systematic positions of the families to which the species belong.

There are obvious differences in genome size among the four species of Saxifragales. These genome size variations are mainly due to length differences in spacers rather than differences in coding genes or introns (Table 1). Interestingly, the sizes of these genomes are negatively correlated with the observed substitution rates (Figure 5). There are strong positive correlations between the numbers of substitutions, repeats and indels in *Cephalotaxus* [60] and Araceae [61], and repeats may play a more important role in sequence divergence [62]. The higher substitution rates found in the genomes of *Paeonia obovata* and *S. sarmentosum* imply that their genomes are more relaxed to changes, giving rise to the loss of some “non-essential” sequences and reducing genome sizes. The reduction of genome size results in saving energy in a cell and promoting the efficiency of replication with lower costs [63-65]. According to this hypothesis, small genomes are more likely to be derived. 

Species with short generation times usually evolve more quickly and exhibit higher substitution rates [66-68]. *Paeonia obovata* and *S. sarmentosum* happen to be perennial herbs of short life histories among Saxifragales species, and the species with the lowest substitution rate, *L. formosana*, is a large tree. Therefore, the length of life cycles of these species would have played a role in the evolution of their chloroplast genomes. 

Studies on the *rbcL* gene have revealed a positive correlation between molecular evolution and species diversity in angiosperms [67]. In Saxifragales weak positive correlation (r=0.64) was observed between the dN values and the species diversity of the five lineages [37]. The lineage represented by *S. sarmentosum* is the most species-rich one which includes ca 1,370 species [69] and has the highest substitution rate. The woody lineage to which *L. formosana* belongs has much few species (ca. 107) and has the lowest dN value. However, there are only 32 species in Paeoniaceae [70] but the dN value of *Paeonia obovata* genome is the second largest. Although such a general tendency may hold true, the species diversity may not be a good indicator of genome evolution rate for specific taxa.

## Conclusion

The chloroplast genomes of Saxifragales species have undergone evolution at the gene level, rather than the genome level, as no significant structural changes are observed among their genomes. However, the examined genomes differ in size, and the detected genome size variations are predominantly due to the length of intergenic spacers, rather than losses of genes and introns, gene pseudogenization or IR expansion or contraction. The genome sizes of these species appear to be negatively correlated with their nucleotide substitution rates. Species with short life histories tend to exhibit smaller genome sizes and higher nucleotide substitution rates. As every species displays its own unique evolutionary history, it is difficult to draw a conclusion without exceptions. It is clear that genome evolution is taxon dependent. 

## Supporting Information

Table S1 ***Sedum sarmentosum*-specific primers used to sequence the complete chloroplast genome.**
(XLS)

Table S2 **A list of genes found in the *Sedum sarmentosum* chloroplast genome.** Intron-containing genes are marked by asterisks (*).
(XLS)

Figure S1 **Alignment of the *rpl22* region in five Saxifragales species.** Codons highlighted in red represent stop codons and codons highlighted in green represent unformed triplet codons. The numbers indicate the positions of nucleotides.
(PDF)

Figure S2 **Alignment of the *rps18* region in five Saxifragales species.** Codons highlighted in red represent stop codons and codons highlighted in green represent unformed triplet codons. The numbers indicate the positions of codons.
(PDF)

Figure S3 **Alignment of the *ycf15* region in five Saxifragales species, *Nicotiana*, and *Vitis*.** The uninterrupted form of *Nicotiana* was used as a reference. Codons highlighted in red represent stop codons and codons highlighted in green represent unformed triplet codons. The nucleotides in red and blue indicate an inversion of the sequence.
(PDF)
