# Supplementary material for: Complete Chloroplast Genome of Sedum sarmentosum and Chloroplast Genome Evolution in Saxifragales
Source: PLoS One. 2013 Oct 18;8(10):e77965. doi: 10.1371/journal.pone.0077965 (PMC3799696; doi:10.1371/journal.pone.0077965)
Supplement: Figure S1 — Alignment of the rpl22 region in five Saxifragales species. Codons highlighted in red represent stop codons and codons highlighted in green represent unformed triplet codons. The numbers indicate the positions of nucleotides. (PDF) [file pone.0077965.s003.pdf]

```
Paeonia obovata          ATG ATA AGG AAA AGA AAG AAA AAC CGG TAT ACA GAA GTA TCC GCT ATA GGT AGC TAT ATG CGC ATA TCA GTT CAA AAA GCA CGA AGG GTA GTT GAT CAG ATT CGT GGA CGT TCC TAC GAG GAA GCT
Liquidambar formosana    ATG ATA AAG AAG AGA AAG AAG AAC CCA TAT ACA GAA GTG TAC GCT TTA AGT CAA CAT ATA TGT ATG TCT GCT CAC AAA GCG CGA AGG GTG GTT GAT CAG ATT CGT GGA CGT TCC TAC GAG GAA ACA
Penthorum chinense       ATG ATA AAG AAG ACA AAG AAG AAT CCA TAT ACA GAA GTA TAT GCT TTA GGG CAA CAT ATA CGT ATG TCG GCT CAT AAA GCG CGA AGA GTA GTT GAT CAG ATT CGT GGA CGT TCC TAC GAG GAA ACA
Sedum sarmentosum        ATG ATA --- --- ATA AAG AAA --- AGG AAT ACT CAA GTA TAT GCT TTA GGT CAA TAT ATC TCA ATG TCT GCT CAC AAA GCA CGA AAA GTA GTT GAT CAG ATT CGT GGT CGT TCT TAT GAG GAA ACA
Heuchera sanguinea       ATG TTG --- --- --- AAG AAA --- AGG AAG ATA GAA GTA TAC GCT TTA GGT CAA CAT ATA TGT ATG TCT GCT CAC AAA GCG CGA AGA GTA GTT GAT CAG ATT CGT GGA CGT TCC TAC GAG GAA ACA
Vitis vinifera           --- --- GAA AAA GAA GAT ATA AAA TTA AAA ACA GAA GTA TAC GCT TTA AGT CAA TCT ATC TCT ATG TCT GCT CAC AAA GCG CGA AGA GTA CTT GAT CAA ATT CTT GGA CGT TCC TAT CGG GAA ACA

                                                                                                                                                          236
Paeonia obovata          CTT ATG ATA CTA GAC CTC ATG CCT TAT CGA GCA CGT TTT CCT ATT TTT AAA TTG CTT TAT TCT GCA GTA GCA AAC GCT CGT CAC AAT ATG GGT TTC CAC --- --- --- --C GTC ATT TGT AAA GCT
Liquidambar formosana    CTT ATG ATA CTA GAA CTC ATG CCT TAT CGA GCG TGT TAT CCT ATT TTT AAA TTG GTT TAT TCT GCA GCA GCA AAT GCT AGT CAC AAT AGG GGT TTC AAC GAA GCA GAT TTA GTC ATT AGT AAA GCC
Penthorum chinense       CTT ATG ATA CTA GAA CTC ATG CCT TAT CGA GCA TGT TAT CCT ATT TTT AAA TTG GTT TAT TCT GCA GCA GCA AAT GCT AGT CAC AAT ATG GGT TTC AAC GAA GCA GAT TTA ATC ATT AGT AAA GCC
Sedum sarmentosum        CTT ATG ATA CTA GAA CTC ATG TCT TAT CGA GCA TGT TAT CCT ATT TTT AAA TTA GTT TAT TCT GCT GCA GCA AAT GCT AGT CAC AAT AAG AAT TTC AAG AAA GGG AAT TTA ATT ATT AGT AAA GTT
Heuchera sanguinea       CTT ATG ATA CTA GAA CTC ATG CCT TAT CGA GCA TGT TAT CCC ATT TTT AAA TTG GTT TAT TCT GCA GCA GCA AAT GCT AGT CAC AAT ATG GGT TTC AAT GAA GCA GAT TTA ATC ATT AGT AAA GCC
Vitis vinifera           CTT ACG ATA CTT GAA CTC ATG CCT TAT CGA GCG TGT TAT CCC ATT TTT AAA TTA ATT TAT TCT GCA GCA GCA AAT GCT AAG CAC AAT ATG GGT TTG AAC GAA GAA TAT TTA GTC ATT ACT AAA GCC

Paeonia obovata          GAA GTA AAT AAG GGT AAT ACC AAG AAA AAA TTA AAA CCG CGA GCC CGG GGA TGT AGT TAT CCG ATA AAA AGA CCC ACC TGT CAC ATA ACT ATT GTA TTA AAA AAT AGA TTC TTA AAT --- --- GAA
Liquidambar formosana    GAA GTC AAC GAA GGT ACT ACC GTG AAA AAA TTA AAA CCT CGG GCT CGA GGA CGT AGT TAT CCG ATA AAA AGA CCC ACT TGT CAT ATA ACT ATT GTA TTG AAA AAT ATA TCC TTA GAT TCA GAT GAA
Penthorum chinense       GAA GTC AAC GAA GGT ACT ACC GTG AAA AAA TTA AAA CCA CGA GCT CGA GGG CGC GGT TTT CCG CTA AAA AGA ACT TCT TGT CAT ATA ACT ATT GTA TTG GAA AAT AGA TCC TTG CTT --- --- AAA
Sedum sarmentosum        GAA GTC AAC GAG GGT ACT ACT GTA AAA AAA TTA AAA CCC CGA GCT CGA GGA CGC GGT TAT CCG ATA AAA CGA CCT AGT TGT CAT ATA ACT ATT GTG TTG AAA GAT ATA TCC TTA ACT --- --- GAA
Heuchera sanguinea       GAA GTC AAT GAG GGT ACT ACC GTG AAA AAA TTA AAA CCT CGA GCT AGA GGA CGT GGT TAT CCG ATA AAA AGG TCC ACC TGC CAT ATA ACT ATT GTA TTG AAA AAT ACA TCC TTA GAT TTA TAT GAA
Vitis vinifera           ACA GTC AAC GAG GGT ACT ACC GTG AAA AAA TTG AAA CCT CGA GCT CGA GGA CGT AGT TAT GCG ATA AAA AGA CCC ACT TGT CAT ATA ACT ATT GTA TTG AAA GAT ACA GCC TTA TAT --- --- GAA

Paeonia obovata          GAA TAT GAA ATT TGG AGA AGA GCA CCT CAA AAT GCA ATT TTA CTA GAA AAA GGA TTA ATA TGA --- --- --- --- --- --- --- --- --- --- --- --- --- --- --- --- --- --- --- ---
Liquidambar formosana    GAA TAT ATC ATA TGA --- --- --- --- --- --- --- --- --- --- --- --- --- --- --- --- --- --- --- --- --- --- --- --- --- --- --- --- --- --- --- --- --- --- --- ---
Penthorum chinense       GAA --- --- --- --- --- --- ATA TCC TCA CTT AGA CGA AGA GTA TGG TGA --- --- --- --- --- --- --- --- --- --- --- --- --- --- --- --- --- --- --- --- --- --- --- ---
Sedum sarmentosum        GAA TAC GAT TAT --- AGA TAT ATA TAA --- --- --- --- --- --- --- --- --- --- --- --- --- --- --- --- --- --- --- --- --- --- --- --- --- --- --- --- --- --- --- ---
Heuchera sanguinea       GAA TAT AGC TTT --- --- --- ATA TGA --- --- --- --- --- --- --- --- --- --- --- --- --- --- --- --- --- --- --- --- --- --- --- --- --- --- --- --- --- --- --- ---
Vitis vinifera           AAA TAT GTC GTA GAA AAC TCT ATC AGG TGT TTA AAA AAA ACC GGA TGG ATA TAT AAA AAG AAG TCC ACA GAT ATT ACG TGT CAT AAT ATG TAT AGT AGT GGG GGA GTA TGG GAC AAA AAA TAA
```

TTG TAA
