# Supplementary material for: Complete Chloroplast Genome of Sedum sarmentosum and Chloroplast Genome Evolution in Saxifragales
Source: PLoS One. 2013 Oct 18;8(10):e77965. doi: 10.1371/journal.pone.0077965 (PMC3799696; doi:10.1371/journal.pone.0077965)
Supplement: Figure S2 — Alignment of the rps18 region in five Saxifragales species. Codons highlighted in red represent stop codons and codons highlighted in green represent unformed triplet codons. The numbers indicate the positions of codons. (PDF) [file pone.0077965.s004.pdf]

```
                      1                                                                                                                                                                                45
Paeonia obovata       ATG GAT AAA CCC AAG CGA CCT CTT CTT AAA TCT AAA CGG CCT TTT CAT AGG CGT TTG CCC CCG AGT CAA TCG GGG GGT CGA ATT GAT TAT AGA AAC CTG AGT TTA ATT AGT CGA TTT ATT AGT GAA CAA GGA AAA
Liquidambar formosana ATG GAT AAA TCC AAG CGA CCC TTT CTT AAA TCC AAG CGA TCT TTT CGT AGG CGT TTG CCC CCG ATC CAA CCG GGG GAT CGA ATT GAT TAT AGA AAT ATG AGT TTA ATT AGT CGA TTT ATT AGT GAA CAA GGA AAA
Penthorum chinense    ATG GAT AAA TCC AAG CGA CCT TTT CTT AAA TCC AAG CGA TCT TTT CGT AGG CGT TTG CCC CCG ATC CAA TCG GGG GAT CGA ATT GAT TAT AGA AAC ATG AGT TTA ATT AGT CGA TTT ATT AGT GAA CAA GGA AAA
Sedum sarmentosum     ATG GAT AAA TCC AAG CGA CCC TTT CTT AAA TCG AAG CGA TCT TTT CGT AGA CGT TTA CCC ACG ATC CAA TCA GGG GAT CGA ATT GAT TAT AGA AAC ATG AGT TTA ATT AGT CGA TTT ATT AGT GAA CAA GGA AAA
Heuchera sanguinea    ATG GAT AAA TCC AAG CGA CCT TTT CTT AAA TCC AAG CGA TCT TTT CGT AGG CGT TTG CCC CCG ATC CAA TCG GGG GAT CGA ATT GAT TAT AGA AAT ATG AGT TTA ATT AGT CGG TTT ATT AGT GAA CAA GGA AAA
Vitis vinifera        ATG GAT AAA TCC AAG CGA CCT TTT CTT AAA TCC AAG CGA TCT TTT CGT AGG CGT TTG CCC CCG ATC CAA TCG GGG GAT CGA ATT GAT TAT AGA AAC ATG AGT TTA ATT AGT CGA TTT ATT AGT GAA CAA GGA AAA

                                                                      58                                                                                                           85                  90
Paeonia obovata       ATA TTA TCT AGA CGA ATG AAT AGA TTG ACC CTG AAA TAA CAA CGA TTA ATT ACT ATT GCT ATA AAA CAA GTT CGT ATT TTA TCT TCG TTA CCT TTT CTT AAT AAT GAG AAA CAA TTT GAA A-A ACC GAG TCG ACC
Liquidambar formosana ATC TTA TCT AGA CGA GTG AAT AGA TTG ACC TTG AAA CAA CAA CGA TTA ATT ACT ATT GCT ATA AAA CAA GCT CGT ATT TTA TCT TCG TTA CCT TTT CTT AAT AAC GAG AAA CAG TTT GAA AGA ACC GAA TCG ACC
Penthorum chinense    ATA TTA TCT AGA CGA GTG AAT AGA TTG ACC TTG AAA CAA CAA CGA TTA ATT ACT ATT GCT ATA AAA CAA GCT CGT ATT TTA TCT TCG TTA CCT TTT CTT AAT AAC GAG AAA CAG TTT GAA AGA ACC GAG TCG ACC
Sedum sarmentosum     ATA TTG TCA AGA CGC GTA AAT AAA TTG ACC TTG AAA CAA CAA CGA TTA ATT ACT ACT GCT ATA AAA CAA GCT CGT ATT TTA TCT TTG TTA CCT TTT CTT AAT AAC GAG AAA CAG TTT GAA AGA ACT GAG TCG ACT
Heuchera sanguinea    ATA TTA TCT AGA CGA GTG AAT AGA TTG ACC TTG AAA CAA CAA CGA TTA ATT ACT ATT GCT ATA AAA CAA GCT CGT ATT TTA TCT TTG TTA CCT TTT CTT AAT AAC GAG AAA CAG TTT GAA AGA ACC GAG TCG ACC
Vitis vinifera        ATA TTA TCT AGA CGA GTG AAT AGA TTG ACT TTG AAA CAA CAA CGA TTA ATT ACT ATT GCT ATA AAA CAA GCT CGT ATT TTA TCT TCG TTA CCT TTT CTT AAT AAT GAG AAA CAA TTT GAA AGA ACC GAG TCG ACC

Paeonia obovata       ACT AGA ACT GCC GGT TTT AGA ACC AAA AAG AGT TTG A
Liquidambar formosana TCT AGA ACT ACT GGT CTT AGA ACC AGA AAT AAA TAG -
Penthorum chinense    GCT AGA ACA ACG GGT CTT AGA CCC AGA AAT AAA TAG -
Sedum sarmentosum     GCT CAA ACA ATA GGT CTT AGA ACT AGA AAT AAA TAG -
Heuchera sanguinea    GCT AGA ACT ACT GGT TTT CGA ACC AGA AAT AAA TAG -
Vitis vinifera        GCT AGA ACT ACT GGT CTT AGA ACC AGA AAT AAA TAG -
```
