# Supplementary material for: Complete Chloroplast Genome of Sedum sarmentosum and Chloroplast Genome Evolution in Saxifragales
Source: PLoS One. 2013 Oct 18;8(10):e77965. doi: 10.1371/journal.pone.0077965 (PMC3799696; doi:10.1371/journal.pone.0077965)
Supplement: Figure S3 — Alignment of the ycf15 region in five Saxifragales species, Nicotiana, and Vitis. The uninterrupted form of Nicotiana was used as a reference. Codons highlighted in red represent stop codons and codons highlighted in green represent unformed triplet codons. The nucleotides in red and blue indicate an inversion of the sequence. (PDF) [file pone.0077965.s005.pdf]

```
Nicotiana tabacum        GTGGAAACGCTTGTTTCTTCCATATTTTGGACC------TTAGCTCCATGGAAGAAT------ATGTTACTGCTGAAACACGGAAGAATTGAAATCTTAGATCAAAACACT------ATGTATGGATGGTATGAACTGCCTA
Liquidambar formosana    GTGGAAACGCTTGTTTCTTCCATATTTTGTACC------TTAGCTCCATGGAACAAT------ATGCTACTGCTGAAACATGGAAGAATTGAAATCTTAGATCAAAACACT------ATGTATGGATGGTATGAACTGCCTA
Paeonia obovata          GTAGGATACGTTTGTTTTTCCATATTTTGGACTTTAGCTTTAGCTCCATGGAACAATATGCTTATGCTACTGCTGAAACATGGAAGAATGGAAATCTTAGATCAAAACACTATGTATATGCATGGATGGTATGAGCTGTCTA
Sedum sarmentosum        GTGGAAATGCTTGTTTCTTCCAAATTTTGGACC------TTAGCTCCATGGAACAAT------ATGCTACTGCTGAAACATGGAAGAATTGAAATCTTGGACT--------------ATGTATGGATGGTATGAACTGCCTA
Penthorum chinense       ----------------------------------------------------------------------------------------------------------------------------------------------
Heuchera sanguinea       GTGGAAACGCTTGTTTCTTCCATATTTTAGACC------TTAGCTCCATGGAACAAT------ATGTTACTGCTGAAACATGGAAGAATTGAAATCTTAGATCAAAACACT------ATGTATGGATGGTATGAACTGCCTA
Vitis vinifera           GTGGAAACACTTGTTTCTTCCATATTTTGGACC------TTAGCTCCATGGAACAAT------ATGCTACTGCTGAAACATGGAAGAATTGAAATCTTAGATCAAAACACT------ATGTATGGATGGTATAAACTGCCTA

Nicotiana tabacum        AACAAGAATTCTTGAACAGCAAACAACCA-----------------------------------------------------------------------------------------------------------------
Liquidambar formosana    AACAAGAATTCTTGAACAGCGAACAACCAAAGCCTATTACTCACTACATCAAAAAATTTCCATTAATGAAAGATGTAAATCCATTGGAAAATCAAAAATACGCATGTCTGATGAAATGGTTGTTGCTATCTGCTCCAATAAC
Paeonia obovata          AACAAGAATTCTTGAACAGCGAACAACCAGAGCCTATTACTCACTACATCAAAAATTTTCCATTAATGAAAGATGTAAATCCATTGGAAAATAAAAAATACGCATGTCTGATGAAATGGTTGTTGCTATCTGCTCCAATAAC
Sedum sarmentosum        AACAAGAATTCTTGAACAGCGAACAACCAGAGCCTATTACTCACTACATCAAAAAATTTC-ATTAATGAAAGATGTAAATCCATTGGAAAATAAAAAATACGCATGTCTGATGAAATGGTTGTTGCTATCTGCTCCAATAAC
Penthorum chinense       ----------------------------------------------------------------------------------------------------------------------------------------------
Heuchera sanguinea       AACAAGAATTCTTGAACAGCGAACAACCAGAGCCTATTACTCACTACATCAAAAAATGTCCATTAATGAAAGATGTAAATCCATTAGAAAATCAAAAATACGCATGTCTGATGAAATGGTTGTTGCTATCTGCTCCAATAAC
Vitis vinifera           AACAAGAATTCTTGAACAGCGAACAACCAGAGCCTATTACTCACTACATCAAAAAATTTCCATTAATGAAAGATGTAAATCCATTGGAAAATCAAAAATACGCATGTCTGATGAAATGGTTGTTGCTATCTGCTCCAATAAC

Nicotiana tabacum        ----------------------------------------------------------------------------------------------------------------------------------------------
Liquidambar formosana    GAATCATTGGTTTAACTGAATA------ACTAAATAAAATAGATAGACCTTTCTCTTCGTCTCAGGTCGATGG------ATCTTCTCAATTGGAAGATACCCTATATGGATAATACACATTCCAGTTGACCAAGCCT-AATT
Paeonia obovata          GAATCGTTGGGTTAACTGAATAAAATA-ACTAAA-AAAATAGATAGACCTTTCTCTTCGTCTCAGGTCGACGG------ATCTTCTCAATTGGAAGATCCCCTATATGGATAATACACATTCCAGTTGACCGAGCCTAAATT
Sedum sarmentosum        AAATCGTTGGTTTAACTGAATA------ACTAAATAAAATAGATCGACCTGAGACGAAGATAAAGGTCGATGGATATGGATCTTCTCAATTGGAAGATCCCCGAGATGGATAATACACATTCCAGTTGACCGAACCC-AATT
Penthorum chinense       ----------------------------------------------------------------------------------------------------------------------------------------------
Heuchera sanguinea       GAATCATTGGTTTAACTGAATA------ACTAACTAAAATAGATAGACCTTTTTCTTCGTCTCAGGTCGATGG------ATCTTCTCAATTGGAAGATCCCCTATATGGATAATACACATTCCAGTTGACCGAGCCT-AATT
Vitis vinifera           GAATCATTGGTTTAACTGAATA------ACTAAATAAAATGGATAGACCTTTCTCTTCGTCTCAGGTCGATGG------ATCTTCTCAATTGGAAGATCCCCTATATGGATAATACACATTCCAGTTGACCGAGCCT-AATT
                                                                          inversion

Nicotiana tabacum        ------------------------------------------------------GTTCAGATATTCACGACCAAGAAGTACTGGATTCTCTTTCGGATAGGCCCTGAAAGGAGAAGGAAGGCTGGAATGCCAACAGGCGTC
Liquidambar formosana    CTAATTGTTTTGTTCCGAAGCAAAGATATCCACGGGGCGGTTCGTCCT------ATTCAGATATTCACGACCAAGAAGTACTGGATTCTCTTTCGGATAGGCCCTGAAAGGAGAAGGAAGGCTGGAATGCCAACAGGCGTC
Paeonia obovata          CTAATTATTTTATTCCGAAGCAAAGATATCCACAGGGTGGTTTGTCCTATTCAGATTCAGATATTCACGACAAATAAGTACTGGATTATCTTTCGGATCGGCCCTGAAAGGAGAAGGAAGGCTGGAATGCTAACAGGCGTC
Sedum sarmentosum        CGAATTGTTTTGTTCAGAAGCAAAGATAGCCACGGGGCGGTTCGCCCT------AGTCAGATATTTACGACCAAGAAGTACTGTA--------CGGATAGGCCCTGAAAGAAGAAGGAAGGCTGGAATGCCAACAGGCGTC
Penthorum chinense       -----------------------------------------------------------------CACGACCAAGAAGTACTGTATTCTTTTTCGGATAGGC------CGGAGAAGGAAGGCTGGAATGCCAACAGGCGTC
Heuchera sanguinea       CTAATTGTTTTGTTCCGAAGCAAAGATATCCACGGGGCGGTTCGTCCT------ATTCAGATATTCACGACCAAGAAGTACTGGATTCTCTTTCGGATAGGCCCTGAAAGGAGAAGGAAGGCTGGAATGCCAACAGGCGTC
Vitis vinifera           CTAATTGTTTTGTTCCGAAGCAAAGATATCCACGGGGCGGTTCGTCCT------ATTCAGATATTCACGACCAAGAAGTACTGGATTCTCTTTCGGATAGGCCCTGAAAGGAGAAGGAAGGCTGGAATGCCAACAGGCGTC

Nicotiana tabacum        TATTATATTGAATTTACCCGATAG
Liquidambar formosana    TATTAT--TGAATTCACCCGACCC
Paeonia obovata          TATTAT--TGAATTCACCCAACCC
Sedum sarmentosum        TAGAAT--TGAATTCACCCGACCC
Penthorum chinense       TA-------GAATTCACTCGACCC
Heuchera sanguinea       TATTAT--TGAATTCACCCGACCC
Vitis vinifera           TATTAT--TGAATTCACCCGACCC
```
